# Developing the National Knowledge Platform in India: a policy and institutional analysis

**DOI:** 10.1186/s12961-018-0283-3

**Published:** 2018-02-20

**Authors:** Veena Sriram, Sara Bennett, V. R. Raman, Kabir Sheikh

**Affiliations:** 1 Center for Health and the Social Sciences, 5841 S. Maryland Avenue, MC 1005, Suite M200, Chicago, IL United States of America; 2 Department of International Health, Johns Hopkins Bloomberg School of Public Health, Room E8140, 615 N Wolfe St, Baltimore, MD 21205l United States of America; 3 WaterAid, 2nd Floor, New Block, RK Khanna Tennis Stadium, DLTA Complex, 1 Africa Avenue, New Delhi, 110029 India; 4 Public Health Foundation of India, Plot No 47, Sector 44, Gurgaon, Haryana India

**Keywords:** India, Knowledge translation, Knowledge translation platforms, Health policy analysis, Evidence-to-policy

## Abstract

**Background:**

The importance of strong engagement between researchers and decision-makers in the improvement of health systems is increasingly being recognised in low- and middle-income countries (LMICs). In 2013, in India, the Ministry of Health and Family Welfare began exploring the formation of a National Knowledge Platform (NKP) for guiding and supporting public health and health systems research in the country. The development of the NKP represents an important opportunity to enhance the linkage between policy-makers and researchers from the health policy and systems research field in India. However, the development process also reflects the highly complex reality of policy-making in the Indian health sector. Our objective is to provide insight into the policy-making process for establishing a health sector knowledge platform in India, and in doing so, to analyse the enabling contextual factors, the interests and actions of stakeholders, and the varying institutional arrangements explored in the development of the NKP.

**Methods:**

We used a qualitative case study methodology, conducting 16 in-depth interviews and reviewing 42 documents. We utilised General Thematic Analysis to analyse our data. Our research team combined perspectives from both outsiders (independent researchers with no prior or current involvement with the policy) and insiders (researchers involved in the policy-making process).

**Results:**

We found that enabling contextual factors, and a combination of government and non-governmental stakeholders with core interests in public health and health systems, were able to gain considerable momentum in moving the idea for the NKP forward. However, complex evidence-to-policy processes in the Indian health sector resulted in complications in determining the right institutional arrangement for the platform. Establishing the appropriate balance between legitimacy and independence, as well as frequent changes in institutional leadership, were found to be additional issues that stakeholders contended with in building the NKP.

**Conclusion:**

As interest in platforms linking health sector policy-makers and researchers grows in LMICs, our findings may allow stakeholders to learn from the Indian experience thus far, and to anticipate some of the facilitators and barriers that could potentially arise in establishing such mechanisms.

## Background

The importance of strong engagement and learning between researchers and decision-makers in the improvement of health systems is increasingly being recognised in low- and middle-income countries (LMICs) [1–3]. A variety of models to facilitate these linkages exist, including the formation of institutionalised platforms that bring together key stakeholders, termed as Knowledge Translation Platforms (KTPs) [1]. KTPs are meant to broker knowledge translation by serving as a space for dialogue and exchange between researchers and decision-makers, informing a cycle of policy-relevant evidence leading to evidence-informed policy [4, 5].

Recently, momentum has been growing in several LMICs to establish, expand and strengthen these platforms [1, 6–8]. In an exploration of existing platforms from ten countries, El-Jardali et al. [6] describe a broadly promising landscape, particularly in the areas of stakeholder exchange efforts and research dissemination. The same study also notes that key challenges exist, including supporting the production of research and the overall financial sustainability of the platforms. Additionally, studies and reflection from Uganda, Zambia and Malawi note similar themes [5, 8, 9]. Building on this approach, stakeholders have advocated for embedding research at all levels of the health system, through the systematic integration of research into decision-making [2, 3, 10]. These types of initiatives are relatively new in the landscape of evidence-to-policy in LMICs, and require systemic shifts in how decision-making is viewed within the health sector.

Evidence from India suggests a growing movement towards strengthening linkages between researchers and decision-makers in the context of health policy and systems research (HPSR) [11, 12]. Stakeholders in the country have expressed concern regarding the disconnect between ongoing health systems research initiatives and the knowledge requirements of decision-makers [13, 14]. Engagement between researchers and decision-makers in the health system is often sporadic; further, such engagements are impeded by a lack of institutional channels for facilitation, gaps in capacities in terms of creation, dissemination and use of policy-relevant knowledge, and an unstable financial environment for supporting policy-relevant research [15, 16].

To address these gaps, in 2013, the Indian Ministry of Health and Family Welfare began to explore the formation of a National Knowledge Platform (NKP) for guiding and supporting public health and health systems research in the country (Box 1). Formalised in 2016, the NKP is meant to systematise dialogue between researchers and research users, to provide funding support for public health and health systems research in priority areas, and to support capacity-building and knowledge management [11, 17]. Other stakeholders involved in the development of the NKP include the National Health Systems Resource Centre (NHSRC), the Indian Council of Medical Research (ICMR), the Public Health Foundation of India (PHFI) and the Alliance for Health Policy and Systems Research (AHPSR). The initiation and formulation of the NKP has been termed an important opportunity within India to enhance the linkage between policy-makers and researchers from the HPSR field in India [11].

The policy-making process for the NKP presented several interesting questions pertaining to both the issues faced by stakeholders in initiating such a platform and the complex reality of policy-making in the Indian health sector [18]. Examining these processes have the potential to provide important insight to Indian stakeholders, and to those from similar LMICs, seeking to strengthen knowledge translation in the health sector. Following a request by AHPSR and PHFI, we conducted a qualitative case study in order to analyse the policy-making process that led to the formalisation of the NKP. This paper draws from that case study, and here, we seek to address the following research questions: (1) What were the contextual features that enabled the establishment of the NKP to emerge as a priority policy? (2) What types of institutional arrangements and functions were discussed and considered by stakeholders? (3) What were the roles and interests of different actors involved in the discussions, how did these differ, and how were conflicting interests resolved?

## Methods

We selected a descriptive analytical case study for our study design due to its utility in gathering rich, context-specific data on contemporary, complex phenomena [19, 20]. A single case, holistic design was utilised where the boundaries of the case were the date of initiation of dialogue between stakeholders around the platform, approximately April 2013, through December 2015, when data collection for this study was completed. Certain contextual factors predating April 2013 have also been included. The formalisation of the NKP followed data collection; for completeness, we have included relevant factual information from 2016 where necessary.

### Conceptual frameworks and definitions

We viewed policy development through the lens of the policy cycle or the ‘stages heuristic’, and aimed to look at both the agenda setting and policy formulation stages; however, we recognised the limitations of this approach particularly in its suggestion of a linear or cyclical process to policy development [19]. We then used the policy triangle to inform the overall design of the study by categorising questions based on the four categories of the framework – actors, context, content and processes [21]. We selected the policy triangle framework as it afforded the flexibility to provide further definition to the various categories in accordance with the objectives of the study. For example, we viewed context as examining broader political and historical factors that were shaping the trajectory of the policy, both in India – within the health sector and in terms of the political environment – and in the field of international public health. In terms of content, we utilised work by Healy et al. [22] to better understand the various types of institutional arrangements in knowledge translation mechanisms. Institutions were seen in this study as “*multifacted, durable social structures, made up of symbolic elements, social activities, and material resources*” [23]. We considered institutional arrangements to be specified formal relationships in the design and construction of an institution, where the relationships between the various organisational components of the institution are defined. In terms of actors, we primarily focused on their stated roles and interests, the latter of which were considered to be real or material, and susceptible to change [24]. We also recognised that interests may be mediated and shaped by underlying stakeholder ideas (causal beliefs) [24], but did not make this a focus of the study. Finally, we incorporated an exploration into the interaction between the components of the triangle such as the ways actors shaped content, context influenced processes, etc.

### Research team

The research team comprised both outsider and insider perspectives. Two authors (VS and SB) led the design and conduct of the study as independent investigators. VS conducted all the interviews. VS and SB finalised the research questions, had access to identified data, and conducted the analysis. The other two authors (VRR and KS), who had been closely involved in the NKP development process, contributed to the initial design of the study, helped identify key stakeholders in the process, provided relevant documents and information, and helped understand and interpret the de-identified analysis.

### Data collection

In-depth interviews with relevant policy actors and a review of documents were utilised in this study.

#### In-depth interviews

Semi-structured interview guides were developed based on the research questions, guiding frameworks and a priori knowledge of the development of the NKP. Questions were designed to be open-ended, and sufficient space was structured within the interview to explore emerging themes and ideas. Individuals who were either involved in the development of the NKP, or policy-makers or researchers who offered perspectives on the linkage between evidence and policy in the health sector in India, but were not involved in the development of the NKP, were eligible to be invited to participate in the study. All sampling decisions were taken by VS and SB, following early discussions with VRR and KS regarding the key stakeholders in the process and a short review of documents. Maximum variation sampling and snowball sampling were used to identify potential participants [25]. Invited participants were contacted by phone or email with a request to participate in the study.

In-depth interviews were conducted with 16 participants between October 2015 and January 2016 (Table 1). Interviews typically lasted approximately 1 hour, with a range of times between 16 minutes to 2 hours 14 minutes. Nine interviews were conducted in-person, five by Skype, and two over the phone. Interviews were audio recorded with the permission of the participants. One participant declined to be recorded, and another interview was not recorded as the it took place via mobile phone. In addition to the other participants, VRR and KS were also interviewed, and the information provided was used as background, informing our understanding of the NKP development process. However, this information is not formally presented within the findings here.

**Table 1:** Interview participants by category

Category	Number of informants
Domestic researchers	8^a^
International researchers	3^b^
Central-level policy-makers	2
State-level policy-makers	1
Former central-level policy-makers	2
Total	16

^a^Four domestic researchers were not involved with the development of the NKP, and were only peripherally aware of its development

^b^One international researcher was not involved in any capacity with the development of the NKP

An information sheet on the study was given to the participant, and each interview guide was adapted to suit the expertise and experience of the given participant. VS conducted all interviews, wrote notes during the interview, and summarised their content in the form of memos. Interviews were transcribed verbatim by two contracted transcribers, and VS de-identified their content. Each participant was assigned a code during analysis. Table 1 presents the organisational affiliations of participants by their current position, as individuals involved in the development of the NKP transitioned to new positions during the period of study.

#### Document review

Document review was used to gather key information on the policy such as content, actors and processes. A total of 42 documents were reviewed for this study, including meeting minutes, communications and published material on the internet, including official organisational websites and grey literature. Documents were identified through discussions with VRR and KS, discussions with participants, and through internet searching.

### Analysis

General Thematic Analysis was used to analyse the data collected in this study, utilising a primarily deductive approach to coding that allowed for some codes to emerge inductively [26, 27]. The codebook for our analysis was developed based on guiding frameworks and on our knowledge of the case. Transcripts and, in the case of unrecorded interviews, notes were combined with accompanying field-notes for coding purposes. VS and SB conducted initial coding by hand, and the process allowed for testing and refinement of codes as well as inductive generation of new codes. VS then used Atlas.ti (Version 1.0.24) to apply this codebook to the remainder of the transcripts and a selection of documents (for example, meeting minutes, policy guidelines or relevant reports). VS and SB continued to iteratively build the codebook during this process.

Codes were then analysed by framework categories – Context, Actors, Processes and further sub-divided Context into Functions, Characteristics and Structures. Two categories emerged from the coding process – Facilitators and Barriers, and Expectations (for the platform). Data under each code were then analysed, and emerging themes were captured in memos. Finally, we sought to combine insider and outsider perspectives of emerging findings through discussion among the authors. We conducted member checking with one participant to clarify particular themes.

### Reflexivity

The use of qualitative methodology in this study was underpinned by a constructivist epistemology, in which there are multiple truths and viewpoints that emerge during the research process, and where the context and biases of the researchers factor into the construction of knowledge from this process [28]. Recognising the combination of outsider and insider perspectives in the research team, all co-authors carefully divided responsibilities from the onset of the project to ensure the independence and integrity of the research process [29]. For example, only de-identified data were used in discussions with VRR and KS; VRR and KS recused themselves from discussions involving themselves or their institution; and VS and SB had full independence regarding analysis where the other authors might be conflicted.

## Results

Our results are organised around our three primary areas of inquiry – contextual factors, institutional arrangements and functions, and actors. Interviews and documents are labelled according to participant codes (for example, I1, I2, etc.) and documents are labelled by their code in the database (for example, D1, D2, etc.).

### Contextual factors

Several contextual factors, beginning from the mid-2000s, prepared the ground for the emergence of the idea of an embedded research platform in India. A chronology of the events related to the development of the NKP is presented in Table 2.

**Table 2:** Chronology of key events in the development of the National Knowledge Platform

Event	Date and location	Policy outcomes
National Rural Health Mission (NRHM) launches	April 2005	Inclusion of budget for external monitoring and evaluation of NRHM
Alliance for Health Policy and Systems Research Nodal Institute is initiated at Public Health Foundation of India (PHFI)	May 2012	PHFI facilitated development of NKP in their capacity as Nodal Institute
Release of ‘Changing Mindsets’ by World Health Organization (WHO) at the Beijing symposium	November 1, 2012, Beijing	Release of WHO’s strategy on health systems researchers that included embedding research within decision-making
Meeting of Indian health systems researchers at the Beijing symposium	November 2, 2012, Beijing	Increasing agreement on need for further engagement between researchers and policy-makers
Consultation on promoting health systems and implementation research in India	April 5, 2013, Ministry of Health and Family Welfare (MoHFW), New Delhi	Announcement by MoHFW of interest and financial support to embed health systems research in MoHFW
Expert consultations on embedding research into decision-making processes (convened by the Alliance)	May 8–9, 2013, Harvard School of Public Health, Boston	Involvement of senior leaders from India in expert discussions on embedding research into decision-making in low- and middle-income countries
Embedding research into health decision-making in India	August 14, 2013, MoHFW New Delhi	Decision to initiate unit within MoHFW Commitment of funds from NRHM
Submission of the National Knowledge Platform (NKP) proposal to MoHFW	January 2014	File for NKP proposal initiated within MoHFW
Health Secretary transferred from MoHFW; new Health Secretary appointed	February 2014	Slowdown in communication from MoHFW regarding NKP status
Change of leadership at National Health Systems Resource Centre (NHSRC); temporary Executive Director appointed	March 2014	File sent to NHSRC or ICMR by MoHFW for comments
National elections – change in government from United Progressive Alliance to National Democratic Alliance	May 2014	
New Permanent Executive Director of NHSRC appointed	May 2014	Emergence of NHSRC as potential base for Secretariat
Health Secretary transferred; new Health Secretary appointed	February 2015	
Director General of Indian Council of Medical Research (ICMR) retires	March 2015	
New Director General ICMR appointed	August 2015	Emergence of ICMR as potential base for Secretariat
MoHFW issues Government Order formalising the establishment of the NKP	August 2016	NHSRC formally proposed as Secretariat; PHFI formally proposed as convener of Scientific Advisory Committee

*ICMR* Indian Council of Medical Research, *MoHFW* Ministry of Health and Family Welfare, *NRHM* National Rural Health Mission, *NHSRC* National Health Systems Resource Centre, *NKP* National Knowledge Platform, *PHFI* Public Health Foundation of India

#### Existing evidence-to-policy linkages in the health sector

Stakeholders described the utilisation of evidence in the health sector as unsystematic, unpredictable and fragmented. Participants from both the government and research communities noted that there were no systematic mechanisms to link policy and research in the health sector within government, and between government and research institutes (I9, I11, I20, I18). Therefore, underlying the formation of the NKP was a seemingly strong desire to evolve a mechanism that would enable such linkages (I1, I9, I18, I2, I18, I11 D1, D3).

Many participants described an inadequate funding for HPSR in India (I10, I11, I3, I17, I18, I19, I20). International organisations were reportedly dominant in the research funding landscape, leading to the dominance of particular agendas and a lack of collaboration amongst Indian research institutes (I8, I10, I11). A central-level policy-maker also commented on the disconnect between the priorities of research funders and the priorities of government (I20).

Despite the lack of a systematic platform for linking evidence to policy in the health sector, participants described existing approaches by which researchers and policy-makers interacted. Several institutions, such as the NHSRC, State Health Resource Centers, the National Institute of Health and Family Welfare, ICMR and NITI Aayog have an aspect of evidence-to-policy in their mandate and, therefore, facilitate some interaction between researchers and policy-makers. Government actors also periodically constitute temporary committees when evidence is required to respond to specific challenges in the health sector (I11, I20). Finally, some participants noted that evidence was only one of several factors that policy-makers considered in their decision-making (I1, I6, I20) and also noted that studies were sometimes commissioned by policy-makers with the specific purpose of justifying a policy approach (I6).

#### Evidence-to-policy mandates

In the 2000s and 2010s, a growing consciousness about the need for stronger linkages between evidence and policy emerged in key government institutions. The initiation of the National Rural Health Mission (a large-scale health systems initiative with an orientation towards maternal and child health) in 2005 marked an increased engagement of MoHFW with health systems issues, and helped change the overall ‘character’ of MoHFW (I2, I11, I19). One reason is that, due to the scope of the programme, the experience of implementing NRHM more vividly highlighted the complexity within the system (I2, I19). The availability of research funds within NRHM for external agencies to conduct monitoring and evaluation was also an important factor in the eventual development of the NKP (I1, I6).

Another important step was the development of the National Health Research Policy, and the establishment of the Department of Health Research (DHR) within the MoHFW in 2007 (I2, I16). The responsibility of leading the DHR was given to the ICMR Director General. The DHR was established to be a knowledge partner to MoHFW, and a Knowledge Management Policy was further articulated, stating that DHR must play a role in strengthening the link between research and policy by enhancing collaboration between various actors in the policy-making and research communities (D39). Further plans to establish a National Knowledge Management Forum were also considered (and mentioned in the 2011–2012 DHR Annual Report), but were not implemented (I2, I6, D16, D39). One participant noted that a reason for the delay was due to conflicting viewpoints within government on funding allocations for the platform (I6).

#### National sovereignty and self-determination

One domestic researcher noted that the issue of national sovereignty and self-determination in policy-making was evolving in the 2000s, and this emphasis on self-determination played a role in shaping the character of emerging health systems initiatives and institutions in India (I6).

“*…the deeper idea is to set up one that can be sustained and that can be meaningful and is owned by natives and helps the researchers and the research community....*” (Domestic Researcher)

However, one participant disagreed, indicating that the role of international donors in Indian health policy-making had been exaggerated, and disagreed with the notion that stronger notions of self-determination facilitated the development of the NKP. Other participants raised this theme less explicitly, particularly in terms of discussing the need for an indigenous funding mechanism for HPSR in the country (I1, I16, I18).

#### Global attention and coordination around health systems research and evidence-informed policy-making

Participants noted that there was increased international attention to health systems research due to the global health systems research symposia initiated in 2010 and held biennially thereafter (I8, I14, I15). Participants felt that the seemingly ‘prominent’ role played by Indian stakeholders in these meetings resulted in some transference of the ideas discussed in these fora to India (I2, I8). For the 2012 meeting, the Secretary of the MoHFW engaged NHSRC and WHO India in organising and funding travel for a group of Indian delegates to the symposium in Beijing (I1, I11). One participant noted that, as a result of their participation, these researchers took a more active role in strengthening the dialogue around evidence-informed policy-making within India (I15).

### Stakeholder roles and interests

A summary of the key actors, and their roles, is provided in Table 3. The key stakeholders involved in this process appeared to emerge from two ‘clusters’, namely those Indian stakeholders working closely with AHPSR (in this case, PHFI), and the government-affiliated stakeholders actively engaged on research activities with MoHFW (in this case, NHSRC and ICMR). Other government and non-government stakeholders were involved sporadically. The interests and positions of these actors regarding the NKP took shape from 2010 and converged in 2013, facilitating the emergence of the NKP proposal.

**Table 3:** Key stakeholders involved in the development of the National Knowledge Platform

Institution	Type of institution	Year direct involvement began	Role
Department of Health and Family Welfare, Ministry of Health and Family Welfare	Union Government	2013	- Initiated formal discussions around strengthening linkages between policy-makers and health systems research institutes - Sanctioned funds from existing National Rural Health Mission research funding to support a mechanism to strengthen interface between researchers and policy-makers
Public Health Foundation of India	Domestic technical and academic body, initiated as a public–private partnership	2013	- Facilitated meetings between MoHFW and health systems research institutes in their role as Nodal Institute for the AHPSR in India - Led proposal development for the NKP following expression of interest from MoHFW for a mechanism to improve interface between researchers and policy-makers - Was initially proposed as secretariat of NKP; in later iterations, retained convenorship of the Scientific Advisory Committee
Alliance for Health Policy and Systems Research	Initiative of multi-lateral agency (WHO), international	2013	- Promoted and facilitated new WHO global strategy on embedding research in decision-making in LMICs - Identified five institutions in LMICs, including PHFI, to serve as Nodal Institutes for health systems research - Facilitated and provided support for policy discussions to develop the NKP from 2013 onwards
National Health Systems Resource Centre	Technical support institution under National Health Mission, MoHFW	2013	- Involved in early discussions regarding the need for a knowledge translation platform - Provided informal feedback to PHFI, and formal feedback to MoHFW, on NKP functions and structures in 2013 and 2015 - In early 2015, became proposed secretariat for the platform; role later formalised through Government Order
Indian Council of Medical Research	Apex body for biomedical and health research in India, funded by the Union Government of India	2015	- Provided formal feedback to MoHFW on NKP functions and structures in 2014 - In late 2015, was proposed as possible convener for the platform; role later formalised as member of Steering Committee

*AHPSR* Alliance for Health Policy and Systems Research, *LMICs* low- and middle-income countries, *MoHFW* Ministry of Health and Family Welfare, *NHSRC* National Health Systems Resource Centre, *NKP* National Knowledge Platform, *PHFI* Public Health Foundation of India

#### New WHO strategy on HPSR

The 2012 WHO strategy on HPSR, driven by AHPSR, sought to focus on a more integrated approach, embedding research in decision-making (I11, I14, I15, D2). Participants noted the close involvement of decision-makers, including senior MoHFW officials and researchers from India, in the development of the strategy (I14, I15). Building on this report, AHPSR wanted to pursue projects where they could test the idea of systematically embedding research into decision-making, and believed that India presented an important opportunity due to its perceived momentum in linking evidence-to-policy, the close involvement of senior Indian policy-makers and researchers in the WHO strategy, and the selection of PHFI in 2012 as one of the new AHPSR Nodal Institutes for advancing health policy and systems research in LMICs (I10, I14, I15). The formal relationship with AHPSR gave PHFI legitimacy and credibility in pursuing discussions with MoHFW (I6, I8, I15).

#### Evolving interest in the MoHFW

The inclusion of research funding within NRHM and the formation of NHSRC and DHR illustrated the growing acceptance within MoHFW of the need for stronger ties between researchers and policy-makers. A new Secretary (MoHFW) was appointed in 2012 who was noted to have strong interest in enhancing the role of evidence in policy-making, and had good connections within the HPSR community in India (I10, I15, I19).

In 2013, MoHFW officials were introduced to the idea of a formal mechanism or knowledge platform during a meeting of Indian researchers who had attended the Beijing Global Health Systems symposium in November 2012 (I1, I10, I11). PHFI had been designated as a Nodal Institute for AHPSR in India in 2012 [30], and in this capacity also supported the organisation of the meeting. A key outcome of the meeting was a consensus that a formal mechanism to link researchers and decision-makers should be established. The Secretary (MoHFW) also indicated that MoHFW should play a primary role in funding such a mechanism, utilising existing funds within NRHM earmarked for research on HPSR by non-governmental entities (D3). Participants repeatedly described the leadership of MoHFW as being instrumental in the development of this idea (I10, I11).

“*Well, there was a nascent aspiration which sort of crystallized in that meeting, lets put it that way. In the sense that the idea of having the research community engage much more actively with health systems which was again part of the Beijing spill over; we were already convinced, but the people who went on fellowships to Beijing, came in as enthusiastic new advocates so they also spoke up and the health ministry* [was] *willing to listen. And therefore it was a fairly happy amalgamation of people with convergent perspectives but coming from different directions.*” (Domestic Researcher)

Simultaneously, interest was growing within PHFI around strengthening the linkage between evidence and policy. Between May 2013 and August 2013, PHFI researchers, with strong support from AHPSR, began pursuing the ideas raised at the meeting convened by MoHFW (I6, I10, I11), and developed proposals for a mechanism to link researchers and decision-makers. Ideas were explored with key stakeholders, including MoHFW and leadership at NHSRC (I1, I10, I11, D27, D28).

A second, smaller meeting was held in August 2013, following which PHFI took the lead in following up on the window of opportunity presented by the Secretary (Health & Family Welfare) (I6, I14, I15). PHFI submitted a proposal for the platform, now formally termed the NKP, in December 2013. In early drafts, the NKP was largely administered by PHFI. The Secretary largely agreed with the configuration, and importantly, initiated a file in December 2013. However, the policy process received a setback when the Secretary was transferred in February 2014.

#### Shifting momentum

The changes in leadership at MoHFW between January 2014 and May 2014 resulted in inactivity regarding the NKP. The new Secretary (MoHFW) affirmed his interest in the platform, and a new round of discussions began between MoHFW, PHFI and NHSRC in late 2014 (D31, D32). The leadership at NHSRC had also changed in March 2014, and the new Executive Director became interested in the idea of NHSRC playing a central role in the NKP. By early 2015, NHSRC had emerged as the preferred option for convening the platform, with PHFI still playing a role in organising one sub-committee (D33). However, the Secretary was once again transferred and replaced in February 2015; however, this time, discussions had sufficiently progressed at MoHFW to provide momentum to the policy process.

#### Involvement of ICMR

Periodic discussions between MoHFW, NHSRC and PHFI were held from January to June 2015, but did not result in formal action. A change in leadership at ICMR/DHR in August 2015 appeared to reinvigorate a discussion around the platform. The new leader of ICMR wanted to enhance the role of ICMR and DHR in supporting the linkage between research and policy-making. Once again, the possible leadership configuration of the NKP began to shift as the new ICMR leader believed that ICMR/DHR could play a convening role in the NKP.

### Institutional arrangements and functions

#### Scope and functions of the NKP

One of the most significant changes in the scope of the platform over time was a suggestion by MoHFW in January 2015 to broaden the scope beyond health systems research to encompass public health research. The proposed functions of the NKP have largely stayed intact from the initial stages of policy formulation (D6, D40).

#### Evolving institutional arrangements

**Fig. 1:**
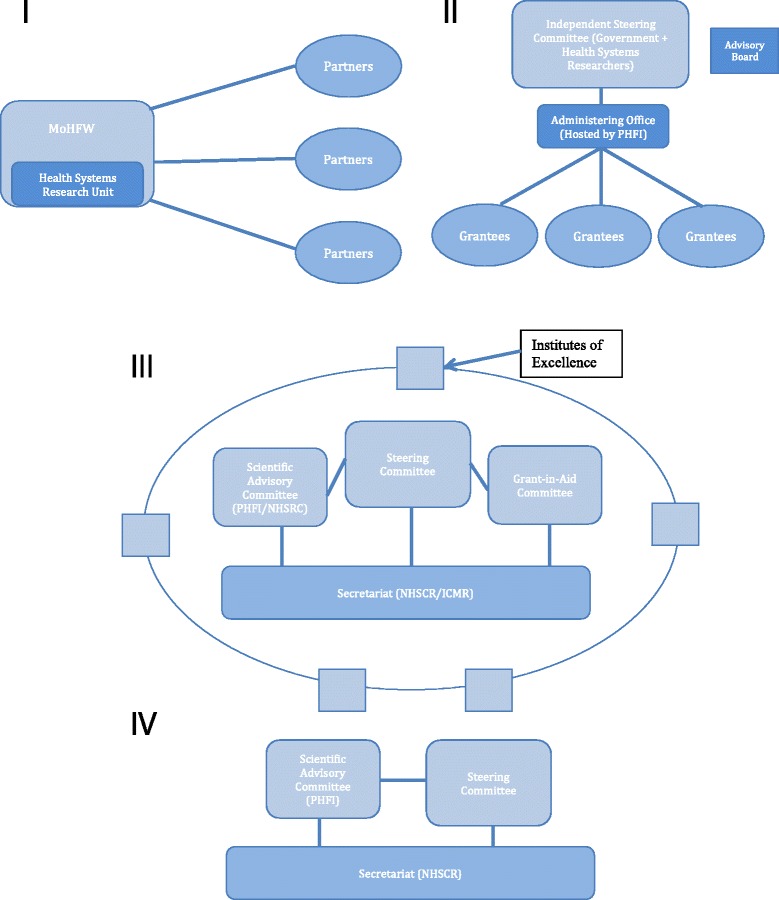
Evolution in institutional arrangement of the National Knowledge Platform in India. I August 2013–Embedded unit that connected to institutional partners (Meeting Minutes, August 14 2013, ‘Embedding Research into Health Decision Making in India’, Nirman Bhavan, New Delhi, India). II December 2013 – Independent platform funded by MoHFW (NKP Proposal, Public Health Foundation of India). III June – December 2015 – Independent platform funded by MoHFW with different leadership configurations (Draft Government Order, National Knowledge Platform June 2015). IV August 2016 – Proposed NKP structure in final Government Order (Final Government Order, August 1 2016, MoHFW)

The arrangement of the proposed platform evolved from the early discussions in mid-2013, to its current format (Fig. 1).

#### Embedded unit

Early on, some stakeholders felt that an embedded unit within MoHFW could ensure traction of research with decision-makers (I1, D3).

“*…it is necessary for the people working with actual policy development, programme development, monitoring, implementation, to be concerned with these issues….evidence-based should not be seen as something somebody outside the Ministry develops to which I have no relation and that it is free for me to either keep or reject. It seems to me that if NRHM funded this, and had these embedded researchers in the Department, there would be much more of, you know, recognition and much more identification with the work they were doing…There’s a place for independent research; there is also a place for some research activities happening out in the Ministry.*” (Former central-level policy-maker)

However, one participant noted that there was a sense within MoHFW that they might not have the capacity to house the structure internally (I6), while another participant mentioned a possible lack of bandwidth to pursue the idea within MoHFW due to other priorities (I1). A further stakeholder argued that the platform would be easier to manage if externally located, compared to the intensive effort required to make permanent changes to the bureaucracy (I15). Stakeholders also expressed a tension between ensuring independence but remaining credible and legitimate. Some researchers noted that the NKP needed to have an ‘arm’s length’ relationship with the Government in order to have independence and objectivity (I8, I10, I11), similar to health sector evidence-to-policy mechanisms in Thailand such as the International Health Policy Programme (I11, I16, I17). One participant shared the following concern of embedding a unit within MoHFW:

“*…Where is the independence of that? Secondly, they may not be able to speak up with boldness to the decision makers. I mean, speaking truth to power is difficult if you are actually getting your salary from there.*” (Domestic Researcher)

Another researcher participant similarly noted:

“*Embedded…you sort of become, you think like the Government. You become more political, you become more administrative and you forget the unbiased nature of research…Embedded also means that your agenda is always the implementation agenda.*” (Domestic researcher)

Conversely, policy-makers and one researcher expressed the need to balance ownership and buy-in from MoHFW and ensure that the findings are appropriately considered and utilised (I1, I11 I16).

“*…the Government never feels comfortable with anything that it cannot control or at least own. So in that sense, it would like at least one of its agencies to be actively engaged…*” (Domestic researcher)

#### Independent platform

In the period between August and December 2013, the idea for the platform evolved from a physically embedded unit to a more independent platform, a conceptualisation proposed by PHFI (D3, D11). Several participants noted that an independent structure with government association would be the ideal home for such a platform. Over the course of 2014 and 2015, the independent nature of the platform stayed intact; however, various additional committees and groups were considered as part of the design, including a Scientific Advisory Committee, an Executive Council (a sub-committee of the Steering Committee), a Grant-in-Aid committee, and Technical Resource Groups. However, some stakeholders reportedly became uncomfortable with the increasing complexity of the NKP as the proposal evolved, and suggested streamlining the structure (D37).

#### Administrative control of independent platform

Once the NKP evolved to be an independent platform outside of MoHFW, possible administrative control ‘wandered’ between PHFI, NHSRC and ICMR (I11). PHFI and NHSRC in particular discussed at length the role of each institution in administering the platform (I8, I9, I10, I11). Some of these changes were due to the leadership changes at these institutions, and the perspectives of incoming leaders regarding the preferred role for their institution in the platform.

Another debate was regarding whether the Chairmanship of the Scientific Advisory Committee should be permanently given to PHFI or rotated across institutions periodically (D5). The proposed arrangement of PHFI permanently retaining the Chairmanship of the Scientific Advisory Committee was facilitated primarily by the early and ongoing interest in MoHFW in having PHFI play that role (D5). However, a few stakeholders outside PHFI expressed concerns about the conflict of interest with PHFI’s involvement given their prominent role in the country as researchers and, therefore, potential beneficiaries of research funding from the platform (I9, D15). Some stakeholders made MoHFW aware of their perceived need for stronger conflict of interest mechanisms. Guiding documents for the Government Order formalising the NKP continued to formally state that individuals involved in any selection process must formally recuse themselves from decision-making where they have a conflict of interest (D8, D42).

Following data collection, a decision was taken by MoHFW for NHSRC to become the Secretariat, and PHFI to convene the Scientific Advisory Committee [11]. ICMR would be involved in the Steering Committee, alongside select government and non-government stakeholders.

#### Key issues in deciding institutional arrangements

As discussions progressed, stakeholders actively debated some key cross-cutting issues.

##### Leadership

The level of leadership of the platform was a key point of negotiation. In early versions of the NKP, the operational leadership of the mechanism was centred at the Secretary level (the highest level in the Indian civil service bureaucracy, at the level of a ministry). One participant reasoned that, while Secretaries may not be involved operationally, their buy-in to the NKP was critical (I16). In other iterations, leadership was pegged at the Additional Secretary level (the second highest level of Indian civil service bureaucracy, at the level of a ministry), and in the final format, was once again elevated to the Secretary level (D40).

##### Engaging other stakeholders

Stakeholders wrestled with how to best engage the numerous institutions involved in evidence-to-policy in the Indian health sector. One participant described the need to work out the mandate and structures, before involving other people and institutions.

“*…first is to establish what the mandate is clearly, and then decide what the functions of this core are and how they are going to be shared around in a complementary manner between the various partners. Then you can reach out to others. If you reach out to others without having the confusion sorted out, then you are not going carry conviction to others, you are only going to disseminate the confusion and either put them off or bring them into a non-productive process.*” (Domestic Researcher)

## Discussion

The formation of a knowledge translation platform focused on public health and health systems research in India was a result of a unique convergence of several factors. At the national level, various policies and reforms in the 2000s and early 2010s, most notably the initiation of NRHM in 2005, primed the Government to support a health sector knowledge platform. National and international context intertwined, with increasing global attention to HPSR in the early 2010s, and in the growing emphasis within WHO and other international health sector stakeholders on strengthening linkages between policy-makers and researchers, two factors strongly articulated in the 2012 WHO strategy on HPSR. Shaped and driven by this context, PHFI and AHPSR emerged in 2012–2013 as ‘institutional entrepreneurs’, working synergistically to establish a new KTP for health policy and systems that would address perceived lacunae in knowledge translation between policy-makers and researchers working in the health sector [23]. Their efforts were facilitated by the presence of highly supportive leadership in MoHFW, which allowed for political and financial commitment for the platform, and supported the leadership of PHFI during the early formulation phase. The increasingly active involvement of other government-affiliated institutions, such as NHSRC and ICMR, indicated that this initiative did indeed have traction within Government, but also simultaneously introduced additional layers of complexity in determining the institutional arrangement due to differing perceptions regarding which stakeholders should and could have administrative control.

The policy process to develop the NKP appears to reflect some of the underlying struggles in developing a KTP of this nature, as well as in policy-making in the Indian health sector more broadly. Our analysis of the development of the NKP suggests three areas that illuminate these issues.

*Complexity of the decision-making architecture*

The complexity of the decision-making architecture in the Indian health sector, and particularly in the realm of linking evidence to policy, was a key challenge in the development of the NKP. The NKP represented a systemic shift in how government and researchers interact and, therefore, necessitated broad stakeholder engagement in its evolution. Yet, the number of institutions in India with some evidence-to-policy mandate, and the frequency of leadership transitions within those institutions, resulted in a complicated policy landscape, one in which policy-making and governance on this issue became challenging.

Such difficulties in governance in the Indian health sector have been widely discussed in the literature and anecdotally. Patel et al. [18] have noted that the ‘inadequate convergence’ of the numerous national- and state-level institutions and programmes has been the largest obstruction to a more efficient health governance approach. Gupta and Khaira [15] also note that the lack of convergence between MoHFW and the Ministry of Women and Child Development has impeded any substantive progress on child malnutrition in India. The experience of developing the NKP adds to the empirical evidence around the complexities of navigating the decision-making space at the central-level in India, and suggests that a protracted, long-term policy negotiation is to be expected in shaping the contours of the programme. Further, given the crowded stakeholder landscape, an evolutionary approach to developing organisational structures, functions and characteristics might work best. In anticipation of sustained negotiations, long-term engagement at multiple levels of the major institutions involved must also take place until the policies are formally notified. In other words, the same heightened level of engagement that was evident in the earliest stages of the NKP policy process could have perhaps continued until formal notification in order to avoid delays.

*Balancing independence and legitimacy*

The objective of establishing a knowledge platform such as the NKP is to ensure that the mechanism is actively used and trusted by the government – this requires that the institution that has administrative control over the mechanism have credibility and legitimacy in the eyes of the government, which might only be possible with an institution that they feel some ownership over. However, ownership actively contends with another objective of these mechanisms – independence – and a key challenge is balancing these two concepts, without diminishing the value of the platform [31]. The variation in proposed institutional arrangements provide a vivid depiction of how stakeholders were contending with, and negotiating, these issues. In their research on health policy analysis institutes in LMICs, Bennett et al. [32] suggest there is “*no single, optimal, institutional distance between a* [health policy analysis institute] *and its target audience*”. This ambiguity actively played out with the NKP, with the locus for convening moving between four institutions, namely MoHFW, PHFI, NHSRC and ICMR.

The Secretary (MoHFW) at the time felt there was a place for an in-house mechanism within the architecture of MoHFW. However, even with committed leadership, establishing such a platform can be logistically difficult, given the array of issues facing policy-makers. Setting up such a mechanism would also require a broader base of support within MoHFW. Both El-Jardali et al. [6] and Berman et al. [9] note the strengths of a Government-based KTP, including an emphasis on addressing government priorities, and closer relationships with the Government. Shroff et al. [7] also found that KTPs with stronger, long-term engagement with government were more effective than those where connections had not been institutionalised. The participants in our study noted similar concerns, and explain the desire of several actors to imbue the structure with strong government ownership.

However, the weaknesses of a government-based approach discussed by El-Jardali et al. [6] and Campos and Hauck [31] were also brought up in this study, such as challenges with researchers negotiating political pressure and maintaining intellectual independence and autonomy. Government-based KTPs also tend to engage with smaller networks of researchers. This could prove to be true in the case of the NKP, as some participants commented on the existing scenario of government tending to engage more trusted researchers and research institutes.

One way to enhance the independence of the mechanism is to establish strong conflict of interest mechanisms within the structure. Such mechanisms were also proposed by Bennett et al. [32] in their study on health policy analysis institutes as a way to protect neutrality and independence. In the case of the NKP, conflict of interest mechanisms were proposed by several participants as a way to ensure that research institutes with administrative control were not given an undue advantage. Other mechanisms can be established to ensure that the independence of the platform is maintained with respect to all stakeholders, including the government.

*Personalised leadership and networks*

Many participants highlighted the primacy of bureaucrats in policy-making in India, and the role of the Secretary (MoHFW) in this study is an excellent illustration of that point. However, as noted in other studies [7, 33], the high turnover rate for these leaders proved to be a major barrier to sustaining momentum. However, beyond affecting the pace of the policy process, the impact of leadership transitions on the process also raises another theme, that of the role of personalised leadership in policy-making in India. In the case of the NKP, the role of individuals in shaping perceptions of their organisation, and in directing policy, was critical and, therefore, the policy development process was vulnerable to those individuals leaving or being transferred. Also of importance were the relationships between individuals, and how those relationships impacted the policy process. For example, strong relationships between individuals at PHFI and MoHFW during the agenda-setting phase helped set the stage for formally initiating the proposal.

The importance of individuals and personal relationships in influencing policy (in the context of health policy analysis institutes) is a ‘double-edged sword’ as influence can wane if those individuals leave the organisation [32]. Koon et al. [3] also note that the quantity, quality, reputation and capacity of organisations and, arguably, individuals, are important factors when assessing the relationship between researchers and decision-makers. Informal relationships between these stakeholders have also been found to be important components of the evidence-to-policy cycle [7, 32, 33]. It is therefore important to consider how to best incorporate the importance of these relationships by helping build trust amongst members, but then ensure that there is adequate institutionalisation of relationships so that the fate of the mechanism is not heavily dependent on personal equations.

### Limitations

We note some limitations to this study. First, policy formulation was not yet complete by the time data collection ended and we were therefore unable to explore the formulation process in totality. Second, MoHFW officials were hesitant to be interviewed in late 2015 as policy discussions were still ongoing, resulting in an inability to incorporate these viewpoints in our analysis. We addressed this issue by speaking with a former MoHFW official and with current central government officials who were all involved in the policy process. Third, our sample was weighted towards participants who were researchers, potentially influencing our analysis; we sought to overcome this challenge by recruiting researchers who worked closely with government on policy development and could therefore provide further insight regarding internal policy discussions. Fourth, some of our participants were peripherally aware of the development of the NKP, and therefore could only comment substantively on the context and their perceptions of the actors involved, and not on the process or the content of the platform.

## Conclusion

The development of the NKP in India represents a unique opportunity to institutionally enhance and strengthen the linkages between researchers and decision-makers in the health sector. Our analysis finds that enabling contextual factors, and a combination of government and non-governmental stakeholders with core interests in public health and health systems, were able to gain considerable momentum in moving this agenda forward. However, the complex evidence-to-policy process in the Indian health sector resulted in complications to determining the right institutional arrangement for the platform. Determining the appropriate balance between legitimacy and independence, and the frequent leadership changes of the key stakeholders involved, also shaped the development of the platform. As more initiatives to strengthen linkages between researchers and policy-makers emerge in LMICs, we hope that the findings of this study support stakeholders in understanding possible trajectories of their efforts, and to anticipate some of the facilitators and barriers that they might experience in developing these mechanisms.

## Box 1: Objectives of the National Knowledge Platform [17]

1) Enabling regular and productive knowledge sharing and dialogue between public health and health systems researchers and research users (policy-makers and implementers) at state and national levels

2) Support for public health and health systems research in priority areas through grant funding in annually identified priority thematic areas – comprising competitive research grant call; commissioned research in response to specific knowledge requirements and conference scholarships

3) Facilitation of research uptake and dissemination

4) Research capacity-building

5) Knowledge management on an open-access platform

## Abbreviations

AHPSR: Alliance for Health Policy and Systems Research
DHR: Department of Health Research
HPSR: health policy and systems research
ICMR: Indian Council for Medical Research
KTP: knowledge translation platforms
LMICs: low- and middle-income countries
MoHFW: Ministry of Health and Family Welfare
NHSRC: National Health Systems Resource Centre
NKP: National Knowledge Platform
NRHM: National Rural Health Mission
PHFI: Public Health Foundation of India
